# A Word to Living Londoners

**Published:** 1895-06-15

**Authors:** 


### A Word to Living Londoners.

### HOSPITAL SUNDAY IN LONDON, JUNE 16th, 1895.

#### A Spirited Effort to Raise £70,000.

A careful inquiry has elicited the fact that the income of the Metropolitan hospitals as a whole is on an average £25,000 a year less than the expenditure. Of this income, in past years from £40,000 to £45,000 has been raised on Hospital Sunday for the Metropolitan hospitals. If this amount can be increased to £70,000 on Hospital Sunday, June 16th inst., the hospitals will be placed on a satisfactory basis for the current year at any rate. The Hospital Sunday Supplement of The Hospital contains many new and interesting features and facts. These include illustrations and diagrams showing the proportion of diseases to population; the diseases from which Londoners suffer; how disease is distributed throughout London; the cost of the work done, and how the money is provided, with a striking illustration entitled "Just Before Bedtime in a Children's Ward." The Archdeacon of London describes this Supplement as "most interesting, able, and timely."

Believing that the facts have only to be realised by Londoners to secure the £70,000 needed on Hospital Sunday, a few gentlemen have intimated to the Editor of The Hospital that they will defray the cost of sending this illustrated Supplement to every well-to-do resident throughout the metropolis. Arrangements are in progress to deliver by special messenger a letter and subscription form, with a copy of the Supplement, to every such inhabitant during Thursday and Friday. Many residents for various reasons may be absent from London, or not able to attend a place of worship on Sunday next. In any case we hope our readers will look out for and welcome this interesting paper, which has important bearings upon their life in London, and that each may be willing to send a cheque for not less than £1 to the Lord Mayor at the Mansion House if unable to give personally, so that the required amount may be forthcoming on Hospital Sunday. No one will deny that the hospitals merit this amount of consideration at the hands of every intelligent and well-to-do citizen.

